# Results of a neonatal hearing screening program in Maceió

**DOI:** 10.1016/S1808-8694(15)30832-6

**Published:** 2015-10-18

**Authors:** Margareth Barbosa de Souza Dantas, César Antônio Lira dos Anjos, Elizângela Dias Camboim, Marcella de Carvalho Ramos Pimentel

**Affiliations:** 1 Student, Speech and hearing therapy, Speech and Hearing Therapist.; 2 Specialist, Otolaryngologist.; 3 Specialist, Speech and Hearing Therapist.; 4 Student, Speech and Hearing Therapist.; Universidade Estadual de Ciências da Saúde de Alagoas - UNCISAL.

**Keywords:** hearing, hearing loss, prevention

## Abstract

Since 1998, after we started the support group for neonatal hearing screening, many other hearing screening programs were held in Brazil. In Alagoas, the first program started in 2003, but none of its results were published. Hearing is paramount for human communication; therefore, childhood hearing loss can impair speech acquisition, emotional, educational and social development. **Aim:** to present the results achieved in a neonatal hearing screening program in Maceió. Materials and Methods: a retrospective analytical study was carried out in order to study the results from tests carried out from September 2003 to December 2006 in a private hospital of Maceió. **Results:** from a total of 2002 newborns, 1,626 fitted the inclusion criteria, 835 (51.4%) males. The hearing screening was considered appropriate in 1416 cases (87.1%), and the most frequently found age was between 16 and 30 days. Finally, 163 (10.0%) children presented risk indicators for hearing loss, and hyperbilirubinemia was the most common indicator. **Conclusions:** statistical results obtained from this hearing screening program show the importance of holding such programs. This study is important because it contributes to further regional or multinational studies.

## INTRODUCTION

Communication skills is a distinctive trace of human existence, being one of the major factors associated with an individual’s well being. Within this context, hearing plays a fundamental role, since it is considered a cornerstone on top of which we build the complex human communication system^1^. Thus, auditory sensorial privation in children impairs not only their communication, but also their receptive and expressive language potential, their learning (reading and writing), their school performance and their social and emotional development^2^.

The first two years of a child’s life have been considered a critical period for the apprehension and development of hearing and language skills. One of the main reasons for that is the fact that the central nervous system has a great plasticity when stimulated earlier on, especially before 6 months of age, increasing the number of nervous connections and, consequently, improving auditory pathway rehabilitation^3^.

The Brazilian Committee on Hearing Loss During Childhood - Comitê Brasileiro sobre Perdas Auditivas na Infância (CBPAI)^4^ recommends the implementation of Universal Neonatal Hearing Screening (UNHS) - Triagem Auditiva Neonatal Universal (TANU). The test must be carried out in all children, from birth until before 4 months of age, and if there is confirmed hearing deficiency, they must be treated before 7 months of age. The main goal of hearing screening is to identify probable cases of hearing disorders that can have medical and/or educational importance^5^.

The implementation of the hearing screening program is viable because of its low cost and the ease of execution under training and supervision^6^. The results obtained with these programs are very relevant, such as, for example, the program of early identification and prevention of hearing disorders of a University in São Paulo. In the present investigation, the population was broken down in two groups: of high and low auditory risk. In the high risk population, during the first year of life it was possible to identify about 2.5% of cases of mild to profound sensorineural hearing loss. The mean age of audiologic diagnosis was 6.6 months of life and 9.8 months for intervention (hearing aid and therapy). In the low risk population, it was possible to identify sensorineural hearing loss in 0.85% of the population^5^.

The test for objective evaluation of the pre-neural peripheral hearing system, with major clinical application is the Otoacoustic Emissions Test (OAE). These are a type of acoustic energy, described by KEMP^7^, generated by the contractility of the outer hair cells during the active mechanism of cochlear function which propagate to the middle ear and external auditory canal where they can be captured^8^.

OAEs are the best screening procedure to detect hearing impairment early on, because it is a fast, non-invasive, easy to interpret and it is a high specificity and sensitivity test which reflects cochlear response without depending on central nervous system maturity^9^. The most clinically used otoacoustic emissions are those by transient stimulus and distortion product, the transient are the most recommended for Neonatal Auditory Screening (NAS) because they are of easy execution and they are able to detect hearing loss above 35dBNA^10^, ^11^. Such technique represents an important progress in the study of hearing loss in normal newborns and in those under risk for hearing impairment^2^, ^7^, ^8^, ^10^, ^12^, ^13^, ^14^, ^15^.

With the growing increase in the number of Universal Neonatal Hearing Screening Programs (UNHSP), there was a major concern in establishing principles and references to control the programs’ efficiency. Principle 1 stresses that every newborn must have access to hearing screening by physiological measure. Principles 2, 3 and 4 approach, diagnosis (before four months of age), intervention (before seven months of age), and auditory monitoring, respectively. Principle number 5 deals on the rights of children and families, guaranteed through informed choice, decision and consent. Principle # 6 makes sure the children’s family’s and test result information be kept confidential. The 7th principle establishes that information systems must be used in order to measure and report the impacts of neonatal hearing screening programs. And finally, principle 8 says that the neonatal hearing programs must provide data to monitor the quality, establish effective cost, mobilize and maintain support in the community, according to the legislation and regulations^16^.

Since 1998, after creating the neonatal hearing screening support group, many hearing screening programs were implemented in our country^11^. In Alagoas, the first program was created in 2003; however publications on this topic are yet to be recorded in the state. Thus, the present paper aims at presenting the results obtained from a neonatal hearing screening program in the city of Maceió-AL, since so far we do not have statistical data that describes such results.

## MATERIALS AND METHODS

We carried out a retrospective, analytical and observational, cross-sectional study in which we analyzed the results of the tests carried out from September of 2003 through December of 2006, present in a data base from a private hospital in the city of Maceió-AL. This study was approved by the ethics committee of our institution under protocol number 666.

In the sample we included the tests from newborns below 3 months of age and took off those non analyzable tests and/or out of the criteria pass/failure used by the Universal Neonatal Hearing Screening Support Group - Grupo de Apoio a Triagem Auditiva Neonatal Universal (GATANU)^11^, which will be detailed later on. Data was collected by analyzing the tests stored in the hospital’s computer. Besides analyzing the results obtained from the Transient Stimulus Otoacoustic Emissions (TSOAE), we analyzed the correlations of these tests with age, gender, and Risk Indicators for Hearing Impairment (RIHI) which were reported and recorded.

Hearing impairment risk indicators identified in our study were: family history of congenital hearing impairment; congenital infection (syphilis, toxoplasmosis, rubella, cytomegalovirus, herpes and AIDS); consanguinity, skull-facial anomalies - including morphological alterations in the ear pinna and external auditory meatus; birth weight below 1,500 g or small for the gestational age (SGA); hyperbilirubinemia; ototoxic medication - including but not limited to aminoglycosides with or without diuretic agents; bacterial meningitis; Apgar score of 0-4 in the 1st minute or 0-6 in the 5th minute; mechanical ventilation for more than 5 days; syndromes associated with conductive or sensorineural hearing loss; neonates that spend more than 48 hours in the ICU and neonatal infections^16^, ^17^.

The data collected were from newborns submitted to neonatal hearing screening by means of capturing otoacoustic emissions by transient stimulus, using the Capella OAE analyzer in order to analyze cochlear function integrity and to rule out the likelihood of the baby having sensorineural hearing loss. According to GATANU, we considered as a pass criterion, the presence of response above 6 dB in at least 3 frequency bands (the frequencies tested were 1; 1.5; 2; 3 and 4 KHz) with response reproducibility above 50%^11^, ^18^.

Research results were descriptively analyzed through the calculation of averages and proportions. We used the Z test for analysis of independent samples and the Odds Ratio in order to analyze the results obtained in respect of the hearing loss. The significance level used was of 5%.

The data collected was stored and analyzed using Excel 2002® (Microsoft Corporation). We calculated the specific frequencies of variables and mean values; afterwards we created binomial tables with crossovers among the variables studied. The simple logistics regression test was used to compare the proportions, when we assessed whether or not there were associations between two or more variables. The significance level (p value) was used to quantify the possibility of our figures present a random distribution. Values below 5% (p<0.05) were deemed significant. The Odds ratio was used in order to calculate the association strength between two variables, so as to quantify the possibility of a risk factor be associated with the event being studied, with a confidence interval of 95%. We also used the Z test to compare the results classified by age, gender and the incidence of risk factors associated with hearing impairment. In such a case, the 5% (or 0.05) significance level was accepted to reject the null hypothesis.

## RESULTS

**Figure 1:**
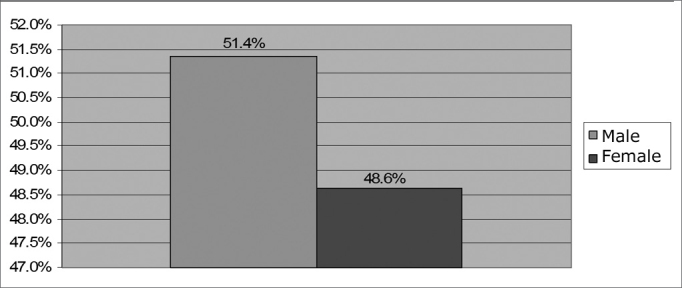
Percentage sample distribution by gender - no legend

**Figure 2:**
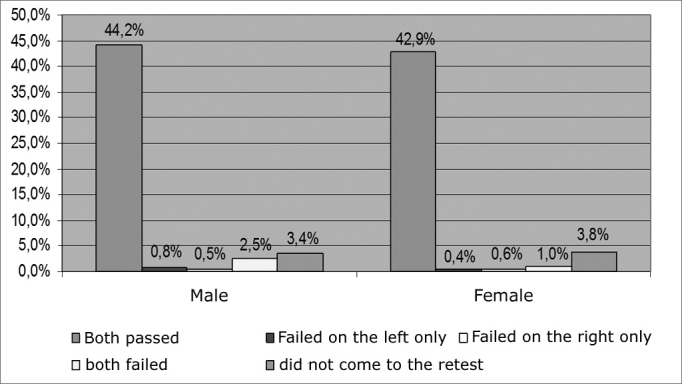
Percentage distribution of NAS results by gender - no legend

From September of 2003 to December of 2006, we carried out NAS in 2002 newborns (NB) in a private hospital in the city of Maceió. Of these, 1,626 matched the inclusion criteria, 791 (48.6%) were girls and 835 (51.4%) were boys (Fig. 1). Among baby boys, 718 (44.2%) passed the NAS; 13 (0.8%) failed on the left ear; 8 (0.5%) failed on the right ear and 40 (2.5%) failed on both ears. Among baby girls, 698 (42.9%) passed; 7 (0.4%) failed on the left ear; 9 (0.6%) failed on the right ear and 16 (1.0%) failed on both ears, as one can see on Fig. 2. we did not observe any significant difference among the incidences associated with gender (p= 0.3436).

**Figure 3:**
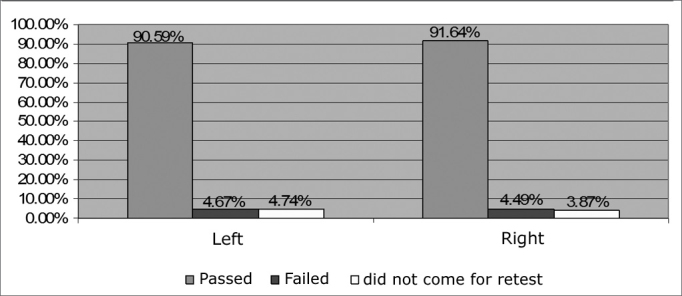
Percentage distribution of NAS results by ear - no legend

In relation to the tests carried out by ear, we noticed that in the right ear there were 1,581 (48.62%) tests, while on the left ear they were 1,580 (48.59%), having that 91 (2.80%) ears were not assessed. Figure 3 shows that on the right ear,

1,488 (91.64%) passed the NAS and 73 (4.49%) failed, on the left ear, 1,472 (90.59%) passed and 76 (4.67%) failed. When the Z test was used, p was equal to 0.2961 indicating a non-significant difference of results in relation to the ears.

**Figure 4:**
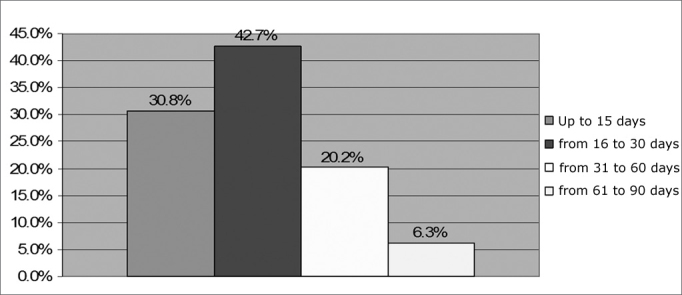
Percentage distribution of NAS by age range - no legend

The most frequent age range at which the NB underwent NAS, as one can see in Figure 4, was the 16 to 30 days interval. This data was significant, since the value of p was of 0.0427.

**Figure 5:**
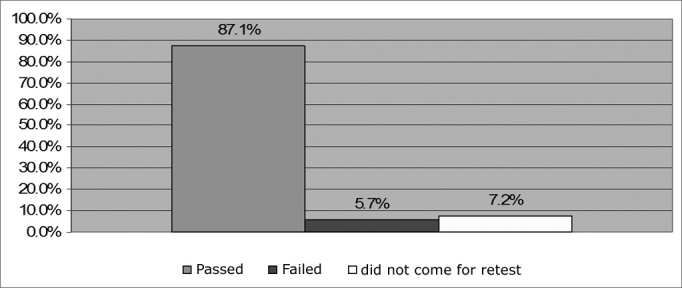
Percentage distribution of NAS carried out at a private hospital in the city of Maceió-AL. - no legend

Of the 1,626 NB seen, 1,315 (80.9%) passed the test and 311 (19.1%) must be referred to a retest. Of these, 194 (11.9%) came and 117 (7.2%) did not come to the retest. With the retesting, we observed that in total, 1,416 (87.1%) NB passed the neonatal auditory screening, while 93 (5.7%) failed (Fig. 5). Of the ones who failed, 20 (21.5%) NB did not have TOAE in their left ears, 17 (18.3%) did not have it in their right ear and 56 (60.2%) did not have TOAE in both ears. The highest incidence of bilateral failure in relation to unilateral ones corroborates previous results present in the literature^19^. As far as gender is concerned, 61 (65.6%) NB were males and 32 (34.4%) were females.

**Figure 6:**
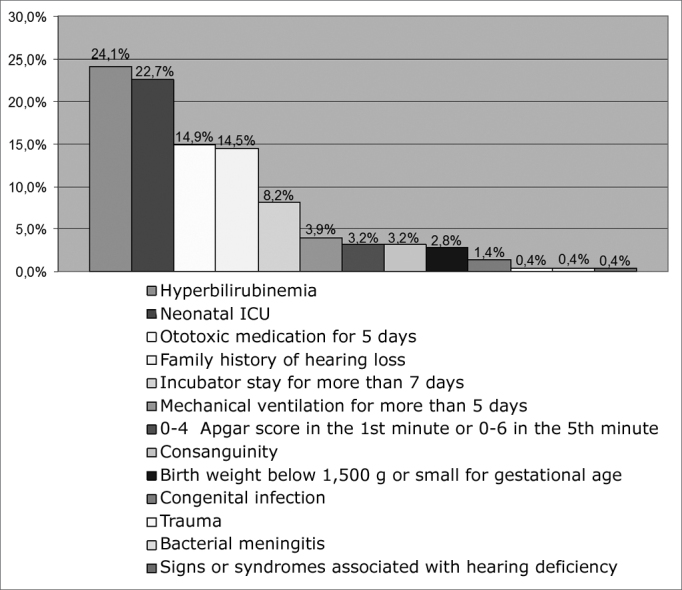
Percentage distribution of risk indicators for hearing impairment - no legend

Our study picked up 163 (10.0%) NB with risk indicators for hearing impairment. Figure 6 shows that the most commonly found RIHI during screening were: hyperbilirubinemia with visible jaundice that not necessarily required complete blood transfusion; followed by neonates who spent more than 48 hours in the neonatal ICU; ototoxic medication; family history of congenital hearing impairment; patient stay in the incubator for more than 7 days; mechanical ventilation for more than 5 days; consanguinity; birth weight below 1,500g or small for the gestational age; congenital infection (syphilis, toxoplasmosis, rubella, cytomegalovirus, herpes and AIDS); bacterial meningitis and skull-facial anomalies. With a mean value of 1.73 risk indicator per person.

Of the NB who failed NAS, 8.6% had some risk indicator for hearing impairment, while 91.4% did not. Of those who had RIHI, 42.9% used ototoxic medication for more than 5 days; 21.40% stayed in the incubator for more than 7 days; 14.30% had family history of congenital hearing impairment; 7.10% had hyperbilirubinemia with visible jaundice - not necessarily needing total blood transfusion; 7.10% used mechanical ventilation for more than 5 days and 7.10% remained in the neonatal ICU for more than 48 hours. Making up a mean value of 1.75 risk indicator per person.

## DISCUSSION

In the present investigation, NBs who had TOAE present were more often males and in the right ear; however, when we applied the Z statistical test, we perceived that these data were not significant. This finding does not match the ones in the literature we studied^6^, ^20^, ^21^, which stresses a higher hearing sensitivity among women and in their right ears, making them have a greater prevalence of otoacoustic emissions.

NAS performance as soon as possible helps optimize intervention, contributing for the child’s development. On Figure 4 we see that most NB underwent NAS in their first months of life, a result which is statistically significant and in agreement with those from other authors^22^, ^23^. This fact may state that health care professionals who work with newborns are attentive to the causes, consequences and the very importance of preventing hearing loss - referring children with hearing loss to the proper professionals such as the hearing therapist and the otorhinolaryngologist in order to carry out proper diagnosis and/or intervention^24^. Early diagnosis in hearing alterations allows for early intervention still in the most critical period and it is ideal to stimulate hearing and speech^25^.

The present study showed that 87.1% passed NAS, 5.7% failed and 7.2% did not come for retest, despite being advised to. It important to stress that to “pass” the test means that at the time of the test, results matched TOAE responses, in other words, showed healthy outer hair cells^26^, ^11^. Throughout children development, acquired hearing loss stemming from: secretory otitis media, infections, ototoxic agents, genetic or traumatic causes can generate permanent hearing impairment^26^.

CBPAI4 states that retest indications should not go beyond 4%. The present investigation found an index of 19.1%, which is significant according to the Z statistical test (p=0.0001), inferring a high number of NB referred for a new assessment. This fact can be justified by the possible presence of noisy breathing, suction act or noise, factors which impair TOAE capture^27^.

Of those NB who had RIHI, Figure 6 shows that hyperbilirubinemia was the most frequently found risk indicator, it is described as the most common cause of hearing deficiency in the newborn, which can damage the inner ear and the central auditory pathways^28^. The use of ototoxic drugs came in second place, as well as consanguinity, genetic hearing loss and the infections diseases that can cause hearing impairment. The same may happen to the NBs who stay in the ICU, for having greater frailty, they may be affected by diseases, procedures or drugs after having been discharged from the nursery.^29^

According to the literature, approximately 50% of the children diagnosed with hearing loss did not have risk indicators for it^27^, ^22^. In the present study, we observed that 91.4% of those who failed the test did not have risk indicators, this stresses the importance of a universal hearing screening program, in other words, for all NB, allowing for equal opportunities for diagnosis and intervention^30^, ^23^.

## CONCLUSION

This study disclosed the statistical results obtained from a neonatal hearing screening program carried out in the city of Maceió-AL, stressing the importance of implementing and maintaining such a program. Moreover, its publication contributes for multinational and/or regional programs.
